# Calculation of exact p-values when SNPs are tested using multiple genetic models

**DOI:** 10.1186/1471-2156-15-75

**Published:** 2014-06-20

**Authors:** Rajesh Talluri, Jian Wang, Sanjay Shete

**Affiliations:** 1 Department of Biostatistics, The University of Texas MD Anderson Cancer Center, Houston, TX, USA; 2 Department of Epidemiology, The University of Texas MD Anderson Cancer Center, Houston, TX, USA

**Keywords:** Genetic association, Multiple testing, Cochran-Armitage trend test, Genetic models, Exact p-value

## Abstract

**Background:**

Several methods have been proposed to account for multiple comparisons in genetic association studies. However, investigators typically test each of the SNPs using multiple genetic models. Association testing using the Cochran-Armitage test for trend assuming an additive, dominant, or recessive genetic model, is commonly performed. Thus, each SNP is tested three times. Some investigators report the smallest p-value obtained from the three tests corresponding to the three genetic models, but such an approach inherently leads to inflated type 1 errors. Because of the small number of tests (three) and high correlation (functional dependence) among these tests, the procedures available for accounting for multiple tests are either too conservative or fail to meet the underlying assumptions (e.g., asymptotic multivariate normality or independence among the tests).

**Results:**

We propose a method to calculate the exact p-value for each SNP using different genetic models. We performed simulations, which demonstrated the control of type 1 error and power gains using the proposed approach. We applied the proposed method to compute p-value for a polymorphism *eNOS -786T>C* which was shown to be associated with breast cancer risk.

**Conclusions:**

Our findings indicate that the proposed method should be used to maximize power and control type 1 errors when analyzing genetic data using additive, dominant, and recessive models.

## Background

Genome-wide association studies (GWAS) and candidate gene association studies are commonly performed to test the association of genetic variants with a particular phenotype. Typically, hundreds of thousands of single-nucleotide polymorphisms (SNPs) are tested for association in these studies. Associations between the SNPs and the phenotypes are determined on the basis of differences in allele frequencies between cases and controls [1]. Several statistical methods have been proposed to control the family-wise error rate (FWER) for multiple comparison testing.

A simple approximation can be used to obtain a FWER of *α* by utilizing the Bonferroni adjustment [2] of $\alpha^{*}=\frac{\alpha }{n}$ and using α* as the threshold for significance for each test. Bonferroni adjustment tends to be conservative when the tests are correlated. In genetic association studies, the SNPs being tested are typically in linkage disequilibrium (LD), which leads to correlation among the tests. An alternative approximation to the Bonferroni adjustment is Sidak’s correction [3,4], $\alpha^{*}=1-{\left(1-\alpha \right)}^{\frac{1}{n}}$ which assumes independence among tests. Conneely and Boehnke [5] proposed a correction that does not assume independence among tests but assumes joint multivariate normality of all test statistics. Other methods to control the FWER include using the false discovery rate (FDR) [6,7].

In genetic association studies, three genetic models--additive, dominant, and recessive--are generally used to test each SNP using the Cochran-Armitage (CA) trend test [8-12]. In association studies the true underlying genetic model is unknown. Some investigators report the smallest p-value obtained from the three tests corresponding to the three genetic models. However, such a procedure inherently leads to an inflated type 1 error rate. Also, FDR-based methods to control FWER are not applicable in this situation because the hypotheses are highly correlated, as the same SNP is tested using different genetic models.

Thus, there is a need to correct for multiple comparisons corresponding to the three genetic tests performed for testing the association of a single SNP. These three tests are not only correlated but also functionally dependent. The standard methods for correcting for multiple testing referred to above are either too conservative or fail to meet the assumptions underlying these methods (e.g., asymptotic multivariate normality, independence among tests). Several approaches have been proposed to account specifically for the multiple comparisons of these three genetic models [13-15]. However, these approaches assume asymptotic tri-variate normality for the additive, dominant and recessive test statistics. While this is a reasonable approximation to correct for multiple comparisons, preliminary investigations regarding the joint distribution of the three test statistics revealed the following insights: 1) the joint distribution of the test statistics is discrete and the grids at which the probability mass function is positive is few and far between; 2) The distribution is highly multimodal in most of the situations, particularly, when the number of cases and controls are different and unimodal only in a handful of situations (e.g. when the number of cases and controls are equal). Therefore, we propose a method to compute the exact joint distribution of the three CA trend tests corresponding to the additive, dominant, and recessive genetic models. We used this joint distribution to compute the exact p-value for testing each SNP using the different genetic models. We performed simulations to demonstrate control of type 1 errors and power gains using the proposed approach. Finally, we applied the proposed approach to assess the significance of the association between a promoter polymorphism, *eNOS-786T>C* and breast cancer risk.

## Methods

Consider a di-allelic SNP locus. The minor (deleterious) allele is labeled as *a,* and the major (normal) allele is labeled as *A*. The deleterious allele *a* is assumed to affect a phenotype *Z*, which takes the values of 0 or 1: *Z* = 1 indicates cases (affected) and *Z* = 0 indicates controls (unaffected). The observed genotype data for the SNP is one of three genotypes (*A*, *A*), (*A*, *a*), or (*a*, *a*). Let *R*_*X*_ denote the number of cases and *R*_*Y*_ denote the number of controls, with *R*_*X*_ + *R*_*Y*_ = *N*. Let *X*_1_, *X*_2_, *X*_3_ and *Y*_1_, *Y*_2_, *Y*_3_ be the number of individuals with genotypes *AA*, *Aa*, and *aa* in cases and controls, respectively. The data can be formulated in a 2 × 3 contingency table, as shown in Table 1. Let *p*_1_, *p*_2_, *p*_3_ be the frequencies of genotypes, *AA*, *Aa* and *aa* in cases and *q*_1_, *q*_2_, *q*_3_ be the frequencies of these three genotypes in controls. The values of *p*_*i*_, *q*_*i*_, *i*=1,2,3 can be estimated from the data as $\;p_{i}=\frac{X_{i}}{R_{X}}$ and $\;q_{i}=\frac{Y_{i}}{R_{Y}}\;$.

**Table 1:** Genotypic counts, parameterizations, and notations for various parameters used in the model formulation

	**Genotype**			
	*AA*	*Aa*	*aa*	Sum
Cases (*X*)	*X*_1_	*X*_2_	*X*_3_	*R*_*X*_
Controls (*Y*)	*Y*_1_	*Y*_2_	*Y*_3_	*R*_*Y*_
Sum	*C*_1_	*C*_2_	*C*_3_	*N*

There have been many approaches in the literature for testing the association between a SNP and disease status. The CA test for trend [8] is generally the most popular and is available in most genetic analysis software packages, such as PLINK [16]. The test statistic for the CA test is as follows:

$$W=\sum_{i=1}^{3}t_{i}\left(R_{Y}X_{i}-R_{X}Y_{i}\right),$$

where the weight, *t*_*i*_, is chosen on the basis of the genetic model considered: additive, dominant, or recessive. The additive model assumes the deleterious effect is linearly related to the number of deleterious alleles. The dominant model assumes the deleterious effect is related to the presence of the deleterious allele. And the recessive model assumes the deleterious effect is related to the presence of both the deleterious alleles. The weights *t* = [*t*_1_, *t*_2_, *t*_3_] corresponding to each of the models are as follows: additive model: *t* = [0, 1, 2], dominant model: *t* = [0, 1, 1], and recessive model: *t* = [0, 0, 1] for genotypes *AA*, *Aa*, and *aa*, respectively. Let the three test statistics corresponding to the additive, dominant, and recessive models be *T*_1_, *T*_2_, and *T*_3_, respectively.

### The joint distribution

Each test statistic, *T*_1_, *T*_2_ and *T*_3_, has an asymptotically normal univariate distribution. Therefore, the p-values for each of these tests can be obtained from their asymptotic distributions. However, reporting the smallest p-value obtained from testing *T*_1_, *T*_2_ and *T*_3,_ individually leads to an inflated type 1 error rate. If the exact joint distribution of the three tests is known, one can compute the exact p-value for the SNP that will account for the multiple correlated tests. We proceed to derive the joint distribution of the three test statistics, *T*_1_ = (*R*_*Y*_*X*_2_−*R*_*X*_*Y*_2_) + 2(*R*_*Y*_*X*_3_ − *R*_*X*_*Y*_3_), and *T*_2_ = (*R*_*Y*_*X*_2_ − *R*_*X*_*Y*_2_) + (*R*_*Y*_*X*_3_ − *R*_*X*_*Y*_3_), and *T*_3_ = (*R*_*Y*_*X*_3_ − *R*_*X*_*Y*_3_). As *T*_3_ = *T*_1_ − *T*_2_, we only need to derive the joint distribution of *T*_1_ and *T*_2_. It is reasonable to assume that the three genotype counts in cases (*X*_1_, *X*_2_, *X*_3_) and the three genotype counts in controls (*Y*_1_, *Y*_2_, *Y*_3_) follow a multinomial distribution, with probabilities (*p*_1_, *p*_2_, *p*_3_) and (*q*_1_, *q*_2_, *q*_3_) respectively. Let $T=\left(\begin{array}{c} T_{1}\\ T_{2} \end{array}\right)$, $X=\left(\begin{array}{c} X_{2}\\ X_{3} \end{array}\right)$ and $Y=\left(\begin{array}{c} Y_{2}\\ Y_{3} \end{array}\right)$. The test statistics can be written as *T* = *AX* + *BY*, where $A=\left[\begin{array}{cc} R_{Y} & 2R_{Y}\\ R_{Y} & R_{Y} \end{array}\right]$ and $B=\left[\begin{array}{cc} -R_{X} & -2R_{X}\\ -R_{X} & -R_{X} \end{array}\right]$. Then the joint probability mass function (pmf) of *T*_1_, *T*_2_ is given by

$$f_{T}\left(T_{1},T_{2}\right)=\sum_{X_{2}=0}^{R_{X}}\sum_{X_{3}=0}^{R_{X}-X_{2}}f_{X}\left(X_{2},X_{3}\right)f_{Y}\left(h\left(X_{2},X_{3},T_{1},T_{2}\right)\right)$$

where *f*_*x*_, *f*_*y*_ are trinomial probability mass functions and *h*(*X*, *T*) = *B*^−1^*T* − *B*^−1^*AX*. The derivation of the joint pmf of *T*_1_, *T*_2_ is detailed in the Appendix. The p-value corresponding to the test statistic (*t*_1_, *t*_2_) can be computed by summing up the probabilities of the test statistics that are equally or less probable than the observed test statistic, which can be written as

$$\mathrm{pvalue}\left(t_{1},t_{2}\right)=\begin{array}{l} \sum_{T_{1}}\sum_{T_{2}}f_{T}\left(T_{1},T_{2}\right)\;\\ \langle T_{1},T_{2}:f_{T}\left(T_{1},T_{2}\right)\leq f_{T}\left(t_{1},t_{2}\right)\rangle \end{array}$$

**Figure 1:**
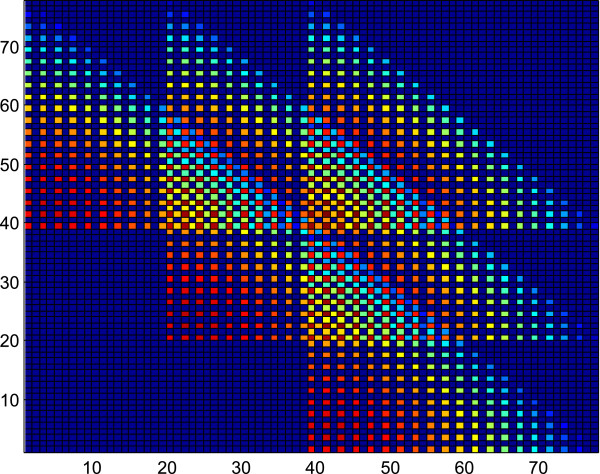
**This figure depicts the probability mass function of the scenario with *R***_***X***_**= 19 and *R***_***Y***_**= 2.** A pattern of six triangles can be visualized.

**Figure 2:**
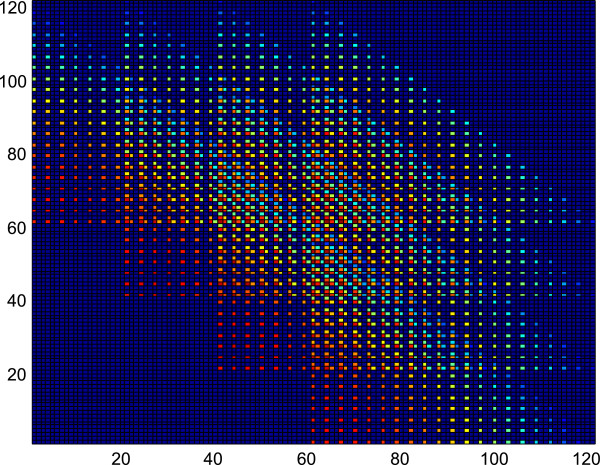
**This figure depicts the probability mass function of the scenario with *R***_***X***_***= *20 and *R***_***Y***_**= 3.** A pattern of ten triangles can be visualized.

**Figure 3:**
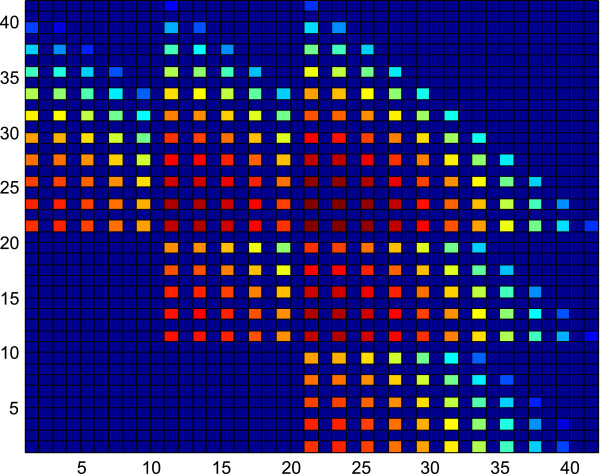
**This figure depicts the probability mass function of the scenario with *R***_***X***_**= 10 and *R***_***Y***_**= 2 and a pattern of six overlapping triangles can be visualized.**

**Figure 4:**
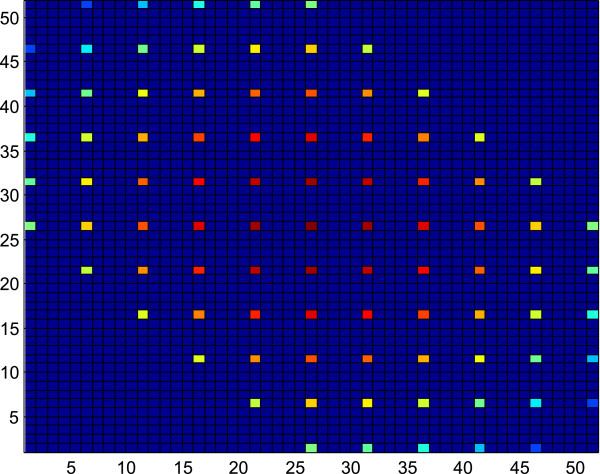
**This figure depicts the probability mass function of the scenario with *R***_***X ***_**= 5 and *R***_***Y ***_**= 5.** A pattern of 21 triangles can be visualized from the figure, where most of the triangles are overlapping completely or partially with one another.

The computation of the p-value using the above formula is nontrivial; however, there are a variety of computational optimizations and parallels to Fisher’s exact test that can be used to drastically reduce the computational complexity (see details in the Appendix). Briefly, the CA trend test statistics form a system of constrained linear Diophantine equations. The computational optimizations presented in the Appendix are based on exploiting the properties of the linear Diophantine equations with trinomial constraints. The solution space of these equations corresponds to the discrete space of nonzero probabilities for the joint pmf. This discrete space has a pattern of overlapping triangles that can be enumerated based on *R*_*X*_ and *R*_*Y*_ counts (See Figures 1, 2, 3 and 4). To reduce the number of computations in the discrete space we first transformed the test statistics to be symmetric. The pattern of overlapping triangles depends on three different scenarios based on the greatest common divisor (GCD) of *R*_*X*_ and *R*_*Y*_: 1. *GCD*(*R*_*X*_, *R*_*Y*_) = 1, 2. *GCD*(*R*_*X*_, *R*_*Y*_) = *R*_*X*_ = *R*_*Y*_ and 3. 1 < *GCD*(*R*_*X*_, *R*_*Y*_) < min(*R*_*X*_, *R*_*Y*_). In scenario 1 the triangles do not overlap, therefore the p-value can be evaluated most efficiently (Figures 1 and 2). In scenario 2 most of the triangles overlap and the discrete space of nonzero probabilities is sparse (Figure 4). In this scenario, we proposed an algorithm to exploit this aspect to calculate the exact p-value more efficiently. Scenario 3 is the most general case which uses the general optimizations of symmetricity and the triangle pattern (Figure 3). The algorithms to compute the exact p-values for each of the scenarios are detailed in the Appendix.

### Simulations

We performed simulations to evaluate the performance of the proposed method and compared our approach with standard approaches used in the literature. All the simulation results were based on 1000 replicate data sets. Each replicate dataset comprised 1000 cases and 1000 controls. The disease status for each data set was obtained using the logistic regression model *logit*(*P*(*Z* = 1)) = *β*_0_ + *β*_1_*X*, where *X* is the indicator for genotype, *Z* is the disease status, *β*_0_ is the intercept, and *β*_1_ is the log odds ratio for the SNP. The genotype data for a SNP were simulated using a minor allele frequency (MAF) of 40% for the null hypothesis and two MAFs of 40% and 20% for the power comparisons. For the type 1 error comparisons, we simulated 1000 replicate datasets from the null hypothesis (i.e., the SNP was not associated with disease status), with *β*_0_ = − 2.5 and *β*_1_ = log (1). For the power comparisons, we simulated 1000 replicate datasets for 40% and 20% MAFs from the alternate hypothesis (i.e., the SNP was associated with disease status) for each of the three scenarios: (1) additive model with odds ratio of 1.2, (2) dominant model with odds ratio of 1.3, and (3) recessive model with odds ratio of 1.3. The methods we compared were as follows: performing only additive analyses (additive-only), performing only dominant analyses (dominant-only), performing only recessive analyses (recessive-only), using the p-value based on reporting the smallest p-value of the three genetic models (min-p), using the Bonferroni correction approach, and using the proposed exact p-value method.

## Results

The type 1 errors based on 1000 replicates from the null hypothesis are shown in Table 2. Analyses based on additive-only, dominant-only, and recessive-only models gave empirical type 1 errors of 0.044, 0.045, and 0.056, respectively, at the 0.05 level of significance. As expected, these models provided good control of type 1 errors because only one genetic model was tested in these analyses. The Bonferroni approach also had a well-controlled, but conservative, type 1 error (0.030 at the 0.05 level of significance). The min-p had a type 1 error of 0.105 at the 0.05 level of significance, which was very liberal and confirmed that the minimum p-value of the three genetic models is not a valid test. Finally, our proposed approach provided good control of the type 1 error (0.047 at the 0.05 level of significance).

**Table 2:** Type 1 error comparisons for different approaches at the 0.05 level of significance for 1000 replicates, each replicate representing a data set containing 1000 cases and 1000 controls

**Method**	**α =0.05**
Additive Only	0.044
Dominant Only	0.045
Recessive Only	0.056
Min-p	0.105
Bonferroni	0.030
Exact p-value	0.047

Min-p: p-value based on reporting the smallest p-value of the three genetic models.

The power comparisons based on 1000 replicates for the SNP data simulated using 40% and 20% MAFs for the three scenarios when the data were simulated using the additive, dominant, and recessive models, respectively, are shown in Table 3. The top and bottom panels of Table 3 depict the results for 40% and 20% MAFs, respectively. The min-p model was excluded from the comparison because of its inflated type 1 error. When the data were simulated using the additive genetic model (column 3, Table 3), and were analyzed using only the additive model, it had the highest powers (0.816 and 0.656 for 40% and 20% MAFs, respectively). However, when the data were analyzed using only the dominant model, the powers were 0.676 and 0.603 for 40% and 20% MAFs, respectively. Also, when the data were analyzed using only the recessive model the powers were 0.588 and 0.306 for 40% and 20% MAFs, respectively. The powers for the additive only analysis were the highest as expected because the true simulation model in this scenario was additive. However, the true model of disease inheritance is generally unknown and one performs analyses using all three genetic models. In this scenario, the proposed exact p-value method had powers of 0.743 and 0.584 for 40% and 20% MAFs, respectively, at the 0.05 level of significance, which were higher than the Bonferroni method which had powers of 0.721 and 0.556 for 40% and 20% MAFs, respectively. Overall, powers of the proposed method were lower than additive model (true simulation model) but higher than those of the dominant-only, recessive-only, and Bonferroni correction approach.

**Table 3:** Power comparisons for different approaches at the 0.05 level of significance for 3 different simulation scenarios using genotypes coded as additive, dominant, and recessive, respectively, for 40% and 20% MAFs

		**Genotype model**		
**MAF**	**Method**	**Additive model**	**Dominant model**	**Recessive model**
		**Odds ratio = 1.2**	**Odds ratio = 1.3**	**Odds ratio = 1.3**
	Additive Only	0.816	0.660	0.410
	Dominant Only	0.676	0.803	0.116
40%	Recessive Only	0.588	0.158	0.589
	Bonferroni	0.721	0.671	0.452
	Exact p-value	0.743	0.726	0.517
	Additive Only	0.656	0.774	0.116
	Dominant Only	0.603	0.823	0.061
20%	Recessive Only	0.306	0.102	0.249
	Bonferroni	0.556	0.715	0.168
	Exact p-value	0.584	0.782	0.197

The results for each panel are based on 1000 replicates, with each replicate representing a data set containing 1000 cases and 1000 controls. MAF: Minor allele frequency.

When the data were simulated using the dominant model (column 4, Table 3), the additive-only, dominant-only and recessive-only analyses had powers of 0.660, 0.803, and 0.158, respectively, for 40% MAF and 0.774, 0.823, and 0.102, respectively for 20% MAF, at the 0.05 level of significance. Once again, as expected, the powers of the dominant-only analysis were the highest because the data were generated using the dominant model. The proposed exact p-value method had powers of 0.726 and 0.782 for the 40% and 20% MAFs, respectively, which were higher than the Bonferroni method which had powers of 0.671 and 0.715 for the 40% and 20% MAFs, respectively. When the data were simulated using the recessive model (column 5, Table 3), the additive-only, dominant-only and recessive-only analyses had powers of 0.410, 0.116, and 0.589, respectively, for 40% MAF and 0.116, 0.061, and 0.249, respectively, for 20% MAF. The proposed exact p-value method had powers of 0.517 and 0.197 for the 40% and 20% MAFs, respectively, which were higher than the Bonferroni method (0.452 and 0.168 for 40% and 20% MAFs, respectively).

We applied the proposed approach to assess the significance of the association between the promoter polymorphism *eNOS -786T>C* and sporadic breast cancer risk in non-Hispanic white women younger than 55 years from a breast cancer study performed by [17]. The study discovered that *eNOS -786T>C* was statistically significant for breast cancer (p=0.017) and included 421 breast cancer cases and 423 cancer free controls. The first panel in Table 4 depicts the genotype counts for TT, CT and CC genotypes in cases and controls for the *eNOS -786T>C*. The second panel in Table 4 reports the p-values for the *eNOS -786T>C* computed using the 5 different approaches: additive-only, dominant-only, recessive-only, Bonferroni and the proposed exact p-value method. The additive-only, dominant-only and recessive-only approaches had p-values of 0.0045, 0.0148 and 0.0313, respectively, and the Bonferroni adjusted p-value was 0.0135. For this SNP, the p-value computed using the proposed exact p-value method was 0.0021, which was more significant than the smallest of the three p-values obtained using the additive-, dominant-, and recessive-only analyses (Table 4).

**Table 4:** **P-values computed using various approaches for association of *eNOS -786T> C *** **with breast cancer**

**Genotype Data for *eNOS -786T> C***			**Method**	**p-value**
	Controls	Cases	Additive Only	0.0045
Total	423	421	Dominant Only	0.0148
TT	203	167	Recessive Only	0.0313
CT	185	200	Bonferroni	0.0135
CC	35	54	Exact p-value	0.0021

## Discussion

In this paper, we proposed a method to calculate the exact p-value for testing a single SNP using multiple genetic models. We recommend using the proposed method to maximize power and control type 1 errors when analyzing genetic data using additive, dominant, and recessive models. The proposed method is robust to model misspecifications and different SNP minor allele frequencies. Furthermore, similar to the computation of Fisher’s exact p-value, the proposed approach does not depend on asymptotic distributions.

In our simulation study, where replicate datasets were simulated using the null hypothesis, we found that the proposed method had well-controlled type 1 error probabilities. In contrast, the method of reporting the smallest p-value of the three genetic models tested had the highest false-positive rate and was found to be invalid. And, as expected, the type 1 error of the Bonferroni correction approach was well controlled but conservative, which typically led to a loss in power for identifying genetic variants.

We also simulated replicate datasets under an alternative hypothesis using the different genetic models: additive, dominant, and recessive. In these simulations, we observed that no single method: additive-only, dominant-only, or recessive-only, had higher power in all three scenarios. Each of these methods had higher power only when the model used to analyze the data was the same as the true model used to generate the data. However, because the true mode of disease inheritance is usually unknown, analyses using all three genetic models are necessary. In general, the Bonferroni correction approach led to higher power than using a model that did not correspond to the true model. The proposed exact p-value method was an improvement over the Bonferroni method. The conservativeness of the Bonferroni method may be due to its inability to account for the functional dependence between the three test statistics. In contrast, our proposed approach accounts for this functional dependence by computing p-values from the joint probability mass function. Finally, we analyzed breast cancer study data in which the polymorphism *eNOS -786T>C*, was found to be significant [17].

The computation time needed to obtain the exact p-value is substantial. The problem is very closely related to Fisher’s exact test, and there are many patterns inherent in the structure of the problem that could be exploited to calculate the p-values more efficiently. In the Appendix, we present several novel optimization techniques to efficiently compute the test statistics in a reasonable time (e.g., approximately 15 min for a 1000 cases and 1000 controls dataset). The software to compute exact p-values is available at http://odin.mdacc.tmc.edu/~rtalluri/index.html.

## Conclusions

In genetic association studies, three genetic models--additive, dominant, and recessive--are generally used to test each SNP using the Cochran-Armitage trend test. Reporting the minimum p-value of the three genetic models leads to inflated type 1 errors. We proposed an approach to compute the exact p-value when genomic data is analyzed using the three genetic models. The proposed approach leads to higher power while controlling the type 1 error.

## Appendix

### Optimization techniques for computing the exact p-value

Recall that *X*_1_, *X*_2_, *X*_3_ and *Y*_1_, *Y*_2_, *Y*_3_ are the number of individuals with genotypes *AA*, *Aa*, and *aa* in cases and controls, respectively, with *X*_1_ + *X*_2_ + *X*_3_ = *R*_*X*_ and *Y*_1_ + *Y*_2_ + *Y*_3_ = *R*_*Y*_. The three genotype counts in cases (*X*_1_, *X*_2_, *X*_3_) and the three genotype counts in controls (*Y*_1_, *Y*_2_, *Y*_3_) follow a multinomial distribution with probabilities (*p*_1_, *p*_2_, *p*_3_) and (*q*_1_, *q*_2_, *q*_3_), respectively. The probability mass function (pmf) of (*X*_*1*_, *X*_*2*_, *X*_*3*_) is $f_{X}\left(X\right)=\frac{R_{X}!}{X_{2}!X_{3}!\left(R_{X}-X_{2}-X_{3}\right)!}p_{1}^{R_{X}-X_{2}-X_{3}}p_{2}^{X_{2}}p_{3}^{X_{3}}$ and the pmf of (*Y*_1_, *Y*_2_, *Y*_3_) is $f_{Y}\left(Y\right)=\frac{R_{Y}!}{Y_{2}!Y_{3}!\left(R_{Y}-Y_{2}-Y_{3}\right)!}q_{1}^{R_{Y}-Y_{2}-Y_{3}}q_{2}^{Y_{2}}q_{3}^{Y_{3}}$. The three test statistics corresponding to the additive, dominant, and recessive models are, *T*_1_ = (*R*_*Y*_*X*_2_ − *R*_*X*_*Y*_2_) + 2(*R*_*Y*_*X*_3_ − *R*_*X*_*Y*_3_) , *T*_2_ = (*R*_*Y*_*X*_2_ − *R*_*X*_*Y*_2_) + (*R*_*Y*_*X*_3_ − *R*_*X*_*Y*_3_), and *T*_3_ = (*R*_*Y*_*X*_3_ − *R*_*X*_*Y*_3_) respectively. As *T*_3_ = *T*_1_ − *T*_2_, we only need to derive the joint distribution of *T*_1_ and *T*_2_. Let $T=\left(\begin{array}{l} T_{1}\\ T_{2} \end{array}\right)$, $X=\left(\begin{array}{l} X_{2}\\ X_{3} \end{array}\right)$, and $Y=\left(\begin{array}{l} Y_{2}\\ Y_{3} \end{array}\right)$. The test statistics can be written as *T* = *AX* + *BY*, where $A=\left[\begin{array}{l} R_{Y}\;2R_{Y}\\ R_{Y}\;R_{Y} \end{array}\right]$ and $B=\left[\begin{array}{l} -R_{X}\;-2R_{X}\\ -R_{X}\;-R_{X} \end{array}\right]$ . We proceed to derive the joint probability mass function of $T=\left(\begin{array}{l} T_{1}\\ T_{2} \end{array}\right)$.

Consider an n-dimensional discrete random vector *G* with pmf *f*_*G*_(). Suppose we have a transformation from *G* → *H*. The pmf *f*_*H*_() of the transformed variables *H* can be expressed as follows: [18]

$$f_{H}\left(H\right)=f_{G}\left(\emptyset^{-1}\left(H\right)\right)$$

This can be extended to the case where the dimensions of *G* and *H* are different, i.e., the transformation from (*X*, *Y*) → *T* is a linear transformation of the form *T* = *AX* + *BY*. The pmf of *T* is given by

$$f_{T}\left(T\right)=\sum_{X}f_{X}\left(X\right)f_{Y}\left(h\left(X,T\right)\right),\;h\left(X,T\right)=B^{-1}T-B^{-1}\mathrm{AX}$$

This can be simplified as:

$$h\left(X,Y\right)=\left(\begin{array}{l} Y_{2}\\ Y_{3} \end{array}\right)=\left(\begin{array}{l} \frac{T_{1}}{R_{X}}-\frac{2T_{2}}{R_{X}}+\frac{R_{Y}X_{2}}{R_{X}}\\ \frac{T_{2}}{R_{X}}-\frac{T_{1}}{R_{X}}+\frac{R_{Y}X_{3}}{R_{X}} \end{array}\right),$$

$$f_{T}\left(T_{1},T_{2}\right)=\sum_{X_{2}=0}^{R_{X}}\sum_{X_{3}=0}^{R_{X}-X_{2}}f_{X}\left(X_{2},X_{3}\right)f_{Y}\left(h\left(X_{2},X_{3},T_{1},T_{2}\right)\right)$$

Computing this pmf on all the possible values of (*T*_1_, *T*_2_) is prohibitively time consuming. Computational optimizations can be used to speed up the computations of the probability mass function. We list several optimization techniques below. The first optimization is to transform the pmf to be symmetric in (*T*_1_, *T*_2_), which reduces the computational burden by half. The original test statistics *T*_1_ and *T*_2_ are *T*_1_ = (*R*_*Y*_*X*_2_ − *R*_*X*_*Y*_2_) + 2(*R*_*Y*_*X*_3_ − *R*_*X*_*Y*_3_) and *T*_2_ = (*R*_*Y*_*X*_2_ − *R*_*X*_*Y*_2_) + (*R*_*Y*_*X*_3_ − *R*_*X*_*Y*_3_), respectively. The joint pmf of (*T*_1_, *T*_2_) is a one-to-one function of the joint distribution of any two orthogonal linear combinations of *T*_1_ and *T*_2_. So if we transform the test statistics *T*_1_ and *T*_2_ into

$$Z_{1}\;=\;\left(R_{Y}X_{3}\;-\;R_{X}Y_{3}\right),$$

$$Z_{2}\;=\;\left(R_{Y}X_{2}\;-\;R_{X}Y_{2}\right),$$

the resulting pmf of (*Z*_1_, *Z*_2_) is a one-to-one function of the pmf of (*T*_1_, *T*_2_). Hence, the p-value obtained will be the same when using (*Z*_1_, *Z*_2_) instead of (*T*_1_, *T*_2_). The resulting pmf of (*Z*_1_, *Z*_2_) can be derived using the same method as with (*T*_1_, *T*_2_).

The next computational optimization is to identify the values that can be taken by (*Z*_1_, *Z*_2_). The number of values (*Z*_1_, *Z*_2_) can take are finite and represented by the solution space of the equations

$$Z_{1}\;=\;\left(R_{Y}X_{3}\;-\;R_{X}Y_{3}\right),$$

$$Z_{2}\;=\;\left(R_{Y}X_{2}\;-\;R_{X}Y_{2}\right),$$

which depends on the values of *R*_*X*_ and *R*_*Y*_. These equations are called linear Diophantine equations and have an infinite number of solutions [19]. But in our case we have multiple constraints on the equations, which reduce the solution space to a finite number of solutions. The constraints are

1. *X*_3_, *Y*_3_, *X*_2_ and *Y*_2_ are integers

2. *X*_3_, *Y*_3_, *X*_2_ and *Y*_2_ ≥ 0

3. *X*_3_ + *X*_2_ ≤ *R*_*X*_

4. *Y*_3_ + *Y*_2_ ≤ *R*_*Y*_

On the basis of these four constraints the solution space can be calculated. While the exact solution space could not be found, it follows a pattern that can be enumerated.

Figure 1 depicts the pmf of the scenario with *R*_*X*_ = 19 and *R*_*Y*_ = 2 where a pattern of six triangles can be visualized from the figure. Similarly, Figure 2 depicts the pmf of the scenario with *R*_*X*_ = 20 and *R*_*Y*_ = 3, where a pattern of ten triangles can be visualized from the picture. This trend can be generalized for all values of *R*_*X*_ and *R*_*Y*_.

Generalizing the above scenario, there are $\left[1+2+\cdot \cdot \cdot +\left(R_{Y}+1\right)=\frac{\left(R_{Y}+1\right)\left(R_{Y}+2\right)}{2}\right]$ triangles for the solution space. In each triangle, there are $\left[1+2+\cdot \cdot \cdot +\left(R_{X}+1\right)=\frac{\left(R_{X}+1\right)\left(R_{X}+2\right)}{2}\right]$ elements that correspond to all possible combinations of *X*_3_ + *X*_2_ ≤ *R*_*X*_. In each triangle, the values of *Y*_3_ and *Y*_2_ are constant and the $\frac{\left(R_{Y}+1\right)\left(R_{Y}+2\right)}{2}$ triangles correspond to all possible combinations of *Y*_3_ + *Y*_2_ ≤ *R*_*Y*_, which make up the whole solution space.

Another important fact is that these triangles may overlap, reducing the solution space, which is depicted in Figures 3 and 4. Figure 3 depicts the pmf of the scenario with *R*_*X*_ = 10 and *R*_*Y*_ = 2 where a pattern of six triangles can be visualized from the figure. The overlap of the triangles can be observed when compared to Figure 1. Figure 4 depicts the pmf of the scenario with *R*_*X*_ = 5 and *R*_*Y*_ = 5 where a pattern of 21 triangles can be visualized from the figure, where most of the triangles are overlapping one another. The additional computational burden is to determine where the solution space triangles overlap and how many triangles are overlapping at a particular location. This is a function of the greatest common divisor (GCD) of *R*_*X*_ and *R*_*Y*_. If *R*_*X*_ and *R*_*Y*_ are co-prime (GCD=1), only three triangles overlap at a single point (*Z*_1_ = 0, *Z*_2_ = 0) which requires no additional computation. When *R*_*X*_ and *R*_*Y*_ are not co-prime, the triangles overlap at multiples of the GCD of *R*_*X*_ and *R*_*Y*_. In this scenario, multiple values of *X*_3_, *Y*_3_, *X*_2,_ and *Y*_2_ contribute to the same (*Z*_1_, *Z*_2_).

In an ideal scenario, the total number of computations required to compute the pmf of (*Z*_1_, *Z*_2_) is $\frac{\left(R_{Y}+1\right)\left(R_{Y}+2\right)}{2}\frac{\left(R_{X}+1\right)\left(R_{X}+2\right)}{2}\approx \frac{R_{X}^{2}R_{Y}^{2}}{4}$, which can be computed in approximately 15 minutes for *R*_*X*_ = 1000 and *R*_*Y*_ = 1000 using a computer with a 3.4-GHz processor and 8 GB of RAM. However, the amount of storage required for the solution space far exceeds the hardware capabilities available. In light of this limitation, computational optimizations should be employed to avoid storing the whole solution space. This limitation leads to three possible scenarios:

1. *GCD*(*R*_*X*_, *R*_*Y*_) = 1

2. *GCD*(*R*_*X*_, *R*_*Y*_) = *R*_*X*_ = *R*_*Y*_

3. *GCD*(*R*_*X*_, *R*_*Y*_) < min(*R*_*X*_, *R*_*Y*_)

### Scenario 1

When *R*_*X*_ and *R*_*Y*_ are co-prime, the triangles only overlap at a single point (*Z*_1_ = 0, *Z*_2_ = 0); therefore, we can independently evaluate each of the possible values of the solution space. The p-value is the probability of obtaining a test statistic at least as extreme as the one observed, so we evaluate the probabilities of each of the possible values of the test statistics one at a time. Hence, the p-value is the sum of all the probabilities of test statistics that are lower than the probability of the observed test statistic. Using this procedure there is no need to store any data, which leads to faster computation of the p-value from the joint distribution.

### Scenario 2

**Figure 5:**
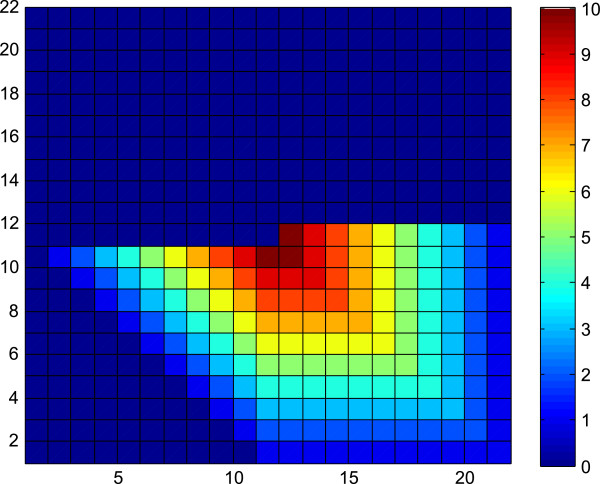
**This figure shows the number of triangles overlapping at each point in the condensed solution space in the scenario with *R***_***X ***_**= 10 and *R***_***Y ***_**= 10, where most of the triangles are overlapping completely or partially with one another.**

When *R*_*X*_ and *R*_*Y*_ are equal, most of the triangles overlap with each other. But a pattern has been observed in this scenario, which is shown in Figure 5, where *R*_*X*_ = 10 and *R*_*Y*_ = 10. As seen in Figure 4, the solution space is very sparse and only requires computation of the colored cells. The possible solution space is spaced *R*_*X*_ apart. So if we condense the possible solution space, the solution space is as shown in Figure 5. Figure 5 shows the number of triangles overlapping at each point in the solution space. Only half of the matrix needs to be computed, as the other half is symmetric. The algorithm to compute the p-value is as follows.

Algorithm:

1. Let *R*_*X*_ = *R*_*Y*_ = *R*. The solution space can then be constrained to a matrix with 2*R* + 1 rows and 2*R* + 1 columns. Let the center of the matrix correspond to the test statistic (*Z*_1_ = 0, *Z*_2_ = 0).2. Now, as we can see from Figure 5, we need to compute the colored cells in quadrants 3 and 4. In quadrant 3, the cells with the same number of overlapping triangles are placed diagonally, and in quadrant 4, they are placed horizontally and then vertically. We exploit the pattern that follows from the same number of triangles overlapping at a particular cell.

3. For *i* = 1: *R* start at (*Z*_1_ = − (*R* − *i*), *Z*_2_ = − 1). Find the possible combinations of *X*_3_, *Y*_3_, *X*_2_ and *Y*_2_ that contribute to the cell corresponding to (*Z*_1_ = − (*R* − *i*), *Z*_2_ = − 1). Compute the probabilities for the cells along the diagonal path in quadrant 3, until *Z*_1_ = 0. Here *X*_3_ and *X*_2_ remain the same; hence, it is trivial to compute the probabilities for each cell.

4. Then in quadrant 4, compute the probabilities for the cells along the horizontal path until *Z*_1_ = *R* − (*i* − 1); here *X*_3_ remains the same and *X*_2*new*_ = *X*_2_ + *Z*_2_.

5. Then continue vertically until *Z*_2_ = 0; here *X*_3_ and *X*_2_ remain the same.

This algorithm reduces the computational burden by computing the possible combinations of *X*_3_, *Y*_3_, *X*_2_ and *Y*_2_ that contribute to all the cells only *R* times, as opposed to computing once for each cell (approximately 4*R*^2^ times).

### Scenario 3

This is the general scenario where *GCD*(*R*_*X*_, *R*_*Y*_) < min(*R*_*X*_, *R*_*Y*_). Several patterns that can be used to reduce the computational burden that could be applied for a particular GCD were found, but these could not be generalized to all the possible situations. We instead use a straightforward approach to determine the p-value for each of the possible solutions for (*Z*_1_, *Z*_2_). The algorithm is as follows:

1. For each possible (*Z*_1_, *Z*_2_) compute the triangles that contribute to this particular point.

2. Add up the probabilities of each of the elements of these triangles to compute the p-value of that particular (*Z*_1_, *Z*_2_).

## Competing interests

We declare that there are no competing interests.

## Authors’ contributions

RT and SS conceived and designed the study. RT implemented the method. RT and JW performed simulations. RT and SS wrote the paper. All authors read and approved the final manuscript.
